# Prognostic nomograms for predicting cause-specific survival and overall survival of stage I–III colon cancer patients: a large population-based study

**DOI:** 10.1186/s12935-019-1079-4

**Published:** 2019-12-27

**Authors:** Zheng Zhou, Shaobo Mo, Weixing Dai, Wenqiang Xiang, Lingyu Han, Qingguo Li, Renjie Wang, Lu Liu, Long Zhang, Sanjun Cai, Guoxiang Cai

**Affiliations:** 1 Department of Colorectal Surgery, Fudan University Shanghai Cancer Center, 270 Dong’an Road, Shanghai, 200032 China; 2 Department of Oncology, Shanghai Medical College, Fudan University, 270 Dong’an Road, Shanghai, 200032 China; 3 Department of Cancer Institute, Fudan University Shanghai Cancer Center, Fudan University, Shanghai, 200032 China; 4 School of Foreign Languages and Cultures, Chongqing University, Chongqing, 401331 China

**Keywords:** Colon cancer, Nomogram, Cause-specific survival, Overall survival, Decision curve analysis

## Abstract

**Background:**

The purpose of this study was to build functional nomograms based on significant clinicopathological features to predict cause-specific survival (CSS) and overall survival (OS) in patients with stage I–III colon cancer.

**Methods:**

Data on patients diagnosed with stage I–III colon cancer between 2010 and 2015 were downloaded from the Surveillance, Epidemiology, and End Results (SEER) database. Univariate and multivariate Cox analyses were used to identify independent prognostic factors, which were used to construct nomograms to predict the probabilities of CSS and OS. The performance of the nomogram was assessed by C-indexes, receiver operating characteristic (ROC) curves and calibration curves. Decision curve analysis (DCA) was used to compare clinical usage between the nomogram and the tumor–node–metastasis (TNM) staging system.

**Results:**

Based on the univariate and multivariate analyses, features that correlated with survival outcomes were used to establish nomograms for CSS and OS prediction. The nomograms showed favorable sensitivity at predicting 1-, 3-, and 5-year CSS and OS, with a C-index of 0.78 (95% confidence interval (CI) 0.77–0.80) for CSS and 0.74 (95% CI 0.73–0.75) for OS. Calibration curves and ROC curves revealed excellent predictive accuracy. The clinically and statistically significant prognostic performance of the nomogram generated with the entire group of patients and risk scores was validated by a stratified analysis. DCA showed that the nomograms were more clinically useful than TNM stage.

**Conclusion:**

Novel nomograms based on significant clinicopathological characteristics were developed and can be used as a tool for clinicians to predict CSS and OS in stage I–III colon cancer patients. These models could help facilitate a personalized postoperative evaluation.

## Background

Colon cancer accounts for more than 1632 deaths per day and has a morality rate of approximately 19.1/100,000, ranking fourth in China and making it the most common malignant digestive tumor [1]. The incidence of colon cancer and patient overall survival (OS) have continued to increase over the past 3 decades worldwide [2]. Patients with colon cancer had a 5-year OS rate of 65.2%, which made colon cancer a serious problem for public health.

The prognosis of colon cancer is associated with the American Joint Commission on Cancer/International Union against Cancer (AJCC/UICC) tumor–node–metastasis (TNM) staging system. According to stages defined by the TNM system, the 5-year stage-specific survival rates are 93.2% for stage I, 82.5% for stage II, and 59.5% for stage III [3]. Nonetheless, patients with stage I–III colon cancer usually have an obviously divergent prognosis because of discrepant genetic and epigenetic backgrounds, even though some colon cancer patients are in the same AJCC stage. Compared with patients with stage IIIa colon cancer, whose 5-year survival rate is 83.4%, patients with stage IIb colon cancer, whose 5-year survival rate is 72.2%, experience severe prognostic events similar to patients with stage IIIb colon cancer, whose 5-year survival rate is 64.1% [4]. Although the TNM staging system is most widely used for prognosis assessment and medical treatments in colon cancer patients, excessive hidden defects still limit its practical application.

Some studies have described that clinicopathological features such as tumor size, the carcinoembryonic antigen (CEA) level, adjuvant chemotherapy, and the log odds of metastatic lymph nodes (LODDS) may also influence colon cancer patients’ survival outcomes [5, 6]. In addition to clinicopathological features, various nonbiological factors were proposed to be included in the patient’s clinical assessment for malignant tumor therapy. For instance, marital status is a significant factor in clinical resolution. Socioeconomic status and insurance status are also important when selecting a treatment strategy. Multiple factors are needed to account for the wide range of variability observed in individual patients. Ignoring these significant prognostic parameters may reduce the accuracy of survival predictions. Thus, a comprehensive prognostic judgment system including clinicopathological and demographic factors is required in clinical practice.

In fact, various prognostic analysis methods have been applied to clinical applications. For instance, microsatellite instability (MSI) or mismatch repair deficiency (dMMR) status is considered the most important biomarker in colon cancer patients [7, 8]. Chromosomal instability (CIN) and CpG island methylator phenotype (CIMP) are also widely accepted as biomarkers for metastasis risk and prognostic analysis [9]. In addition, certain genes and molecules, such as the KRAS gene, the APC gene, the p53 gene, CD44, CD133, and MEK, have been found to be indicators for judging the prognosis of colon cancer patients [10–12]. However, these methods of detection and judgment not only sometimes result in trauma to the patient but also have considerable economic costs. As a convenient and saving graphical interface of a statistical prediction model, nomograms in which various significant variables are combined to predict a specific endpoint have been built by scientists to meet this demand. By integrating these clinical and pathological features, a nomogram simplifies the complicated computational model into a single numerical estimation probability, such as death or disease recurrence, which is tailored to the individual condition. Therefore, a nomogram might be used as a dependable instrument for predicting patients’ survival outcomes and supporting decisions with regard to surgery, surveillance, and adjuvant treatments. Recently, some researchers have reported that the nomogram scoring system has an exceptional capability in predicting prognosis [13, 14]. However, most nomograms used to predict the prognosis of patients with colon cancer, of which the sample size used for development was limited, required a combination of molecular biology tests, which increased the economic burden, time and cost for the patient. This research aimed to develop nomograms that require only clinical features combined with the patient’s socioeconomic status, which is easy to obtain.

The Surveillance, Epidemiology, and End Results (SEER) program provides a profusion of integral information for different cancers from 20 cancer registries that cover ~ 28% of the population. Based on the SEER database, researchers have conducted several studies on the prognosis of cancer [15]. In the present research, information on stage I–III colon cancer was collected from the SEER database to build a nomogram that was intuitive and convenient for predicting the prognosis of colon cancer patients.

## Methods

### Patients selection

**Fig. 1:**
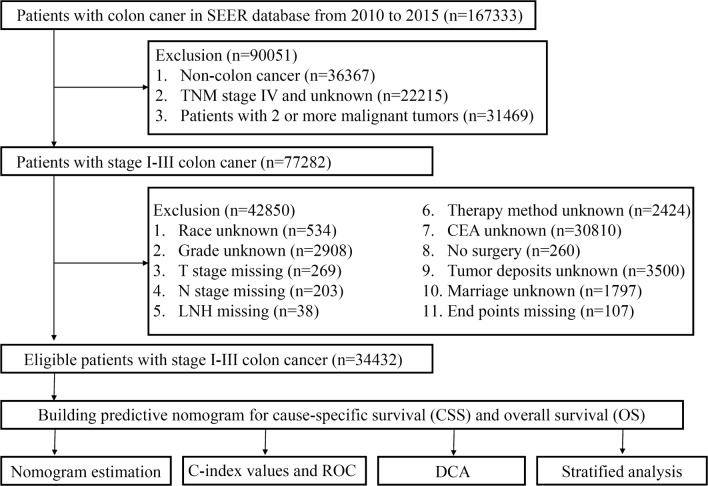
The workflow of establishment of nomograms to predict cause-specific survival and overall survival of patients with stage I–III colon cancer

In this study, a total of 167,333 patients with colon cancer were acquired from the SEER database. The detailed workflow for patient selection is shown in Fig. 1. All colon cancer patients treated with radical surgery between January 1, 2010 and December 31, 2015, were assessed for inclusion in the retrospective analysis. Patients were excluded if non-colon cancer was stated in the pathology report, if they were diagnosed with TNM stage IV or an unknown stage cancer and if they suffered from 2 or more malignant tumors. Eighteen variables were extracted from the SEER program in this study, including race, carcinoembryonic antigen (CEA) level, age, year of diagnosis, sex, adjuvant chemotherapy, histological type, grade, tumor size, number of lymph nodes harvested (LNH), regional nodes positive, LODDS stage, marital status, tumor site, tumor deposit, T stage, N stage, and TNM stage. Patients whose races were recorded as Native American, Asian, Pacific Islander and unknown in the SEER database were assigned to the “other” race category for analysis. Patients without any of these 18 variables were excluded. Patient survival was measured as cause-specific survival (CSS) and OS [16]. Finally, data on 34,432 patients diagnosed with stage I–III colon cancer between 2010 and 2015 were obtained from the SEER database.

### Construction and validation of the nomogram

Univariable and multivariable Cox regression analyses were used to calculate the effect of variables on CSS and OS. The measure of the effect of each variable on CSS and OS is presented as the hazard ratio (HR) and was used to identify independent risk factors. Based on the multivariable Cox regression analyses, two applied nomograms incorporating clinicopathological parameters into the TNM staging system were formulated. The total points in each case of the two survival groups were calculated using the established nomograms, after which Cox regression analysis of the whole cohort was performed using the total points as a parameter. Patients were divided into low- and high-risk groups based on the nomogram risk score and using the median risk score as the cut-off point.

### The concordance index (C-index), receiver operating characteristic (ROC) and decision curve analysis (DCA)

The distinguishing ability of the nomogram was evaluated by the C-index and ROC curve analysis. The C-index was defined as the ratio of all patient pairs predicted to be consistent with the results. The 1-, 3-, and 5-year ROC curves were used to appraise the nomogram’s predictive ability over time. DCA was recently proposed as a fresh method of evaluating predictive models and can be used to visualize the clinical consequences of a treatment method [17]; thus, DCA was carried out to compare the latent profit of the prognostic nomogram in this study.

### Risk stratification based on the novel nomogram

To verify the independent discriminatory ability of the nomogram, this research regrouped all patients into high-, moderate-, and low-risk groups according to the total risk scores. Survival curves for different risk groups were generated using the Kaplan–Meier method and were differentiated using the log-rank test.

### Statistical analyses

R software (version 3.6.0, http://www.r-project.org) was used for all statistical analyses. The R statistical packages “rms”, “survival”, “Hmisc”, “MASS”, and “survivalROC” were used to calculate the C-index, plot the calibration and ROC curves, build the nomogram, and draw Kaplan–Meier curves, while the package “rmda” was used to draw the DCA curves. All statistical tests were 2-sided, and p values < 0.05 were considered statistically significant.

## Results

### Patients’ clinical characteristics and survival outcomes

Data on a total of 34,432 patients with stage I–III colon cancer were retrospectively collected from the SEER database. The patients’ clinicopathological characteristics and 1-, 3-, and 5-year CSS and OS rates are listed in Table 1.

**Table 1:** Patient characteristics and 1-, 3-, and 5-year CSS and OS rates

Characteristics	N	%	CSS			OS		
			1-year (%)	3-year (%)	5-year (%)	1-year (%)	3-year (%)	5-year (%)
Gender								
Female	17,427	50.6	94.5	87.1	82.3	91.7	81.0	72.6
Male	17,005	49.4	95.5	88.5	83.1	92.0	81.4	72.0
Race								
White	26,839	78.0	94.9	88.1	83.5	91.6	81.2	72.6
Other^a^	7593	22.0	95.0	87.1	82.4	92.1	81.4	71.9
Year of diagnosis								
2010–2012	16,262	47.2	94.8	87.5	82.6	91.6	80.8	72.0
2013–2015	18,170	52.8	95.2	87.9	84.5	92.0	81.6	76.8
Age at diagnosis								
< 60	10,602	30.8	98.3	92.8	87.7	97.6	91.1	85.0
≥ 60	23,830	69.2	93.5	85.5	80.5	89.2	76.9	66.8
Tumor site								
Right	19,807	57.5	94.3	86.5	82.0	90.8	78.9	69.9
Left	14,625	42.5	95.9	89.5	83.8	93.2	84.3	75.6
Histological subtype								
AD	31,239	90.7	95.2	88.3	83.3	92.1	81.9	73.0
MAD	2903	8.4	94.2	84.8	80.0	90.4	76.6	68.0
SRCC	290	0.9	78.2	58.5	51.9	74.4	52.7	44.8
Pathologic grade								
Grade I	2585	7.5	97.1	92.3	89.0	94.3	85.9	78.3
Grade II	25,438	73.9	96.0	89.6	84.6	92.9	83.1	74.2
Grade III	5374	15.6	90.5	79.4	73.7	87.1	72.9	63.3
Grade IV	1035	3.0	87.7	74.1	68.3	84.0	67.4	58.3
T stage								
T1	3295	9.6	99.0	97.8	96.5	96.5	92.3	86.6
T2	5901	17.1	97.8	96.0	93.6	94.8	88.7	81.2
T3	20,390	59.2	95.5	88.1	82.9	92.3	81.4	72.4
T4a	3122	9.1	88.4	70.7	60.0	84.4	64.6	51.4
T4b	1724	5.0	84.0	66.6	56.7	80.7	61.4	50.8
N stage								
N0	20,991	61.0	96.9	93.2	90.1	93.7	85.9	78.0
N1	9124	26.5	93.9	83.8	76.8	90.8	78.2	68.1
N2	4317	12.5	87.8	69.7	59.6	84.9	64.7	53.4
Chemotherapy								
No	23,106	67.1	94.0	88.2	84.5	89.8	79.6	70.9
Yes	11,326	32.9	97.0	87.0	79.5	96.0	84.3	75.2
LNH								
< 12	3562	10.3	91.9	82.6	77.3	87.2	73.8	64.1
≥ 12	30,870	89.7	95.3	88.4	83.4	92.3	82.1	73.3
LODDS stage								
LODDS 1	21,981	63.8	96.9	92.9	89.7	93.6	85.6	77.7
LODDS 2	11,376	33.0	93.1	81.4	73.4	90.2	76.0	65.3
LODDS 3	1009	2.9	78.9	54.2	44.7	75.5	49.0	39.3
LODDS 4	66	0.3	46.1	16.8	12.0	43.2	15.8	11.3
Tumor size								
< 4	13,757	40.0	96.9	91.6	87.4	94.0	85.2	76.6
≥ 4	20,675	60.0	93.7	85.2	79.6	90.3	78.5	69.4
CEA								
Negative	22,289	64.7	96.6	91.4	87.5	94.0	85.7	77.9
Positive	12,143	35.3	92.0	80.9	73.7	87.8	73.0	62.0
Tumor deposit								
Negative	32,106	93.2	95.3	88.8	84.0	92.2	82.1	73.3
Positive	2326	6.8	90.3	72.8	63.2	86.8	68.1	56.6
Marriage status								
Married	19,319	56.1	96.4	90.4	85.7	94.0	85.4	77.8
Single	5576	16.2	95.2	86.4	80.8	92.1	79.9	70.8
Separated/Divorced	3769	10.9	95.0	87.1	81.5	92.0	80.9	71.7
Widowed	5768	16.8	89.9	80.3	75.2	84.2	68.7	56.2
TNM stage								
I	7554	21.9	98.4	97.3	95.6	95.6	90.3	83.6
II	13,437	39.0	96.1	90.9	87.0	92.6	83.5	74.9
III	13,441	39.1	92.0	79.3	71.3	88.9	73.9	63.4

*CSS*, cause-specific survival, *OS* overall survival, *AD* adenocarcinoma, *MAD* mucinous adenocarcinoma, *SRCC* signet ring cell carcinoma, *LNH* lymph nodes harvested, *LODDS* log of odds between the number of positive lymph node and the number of negative lymph node, *CEA* carcinoembryonic antigen, *TNM* tumor-node-metastasis

^a^Includes Black, Native American, Asian, Pacific Islander and Unknown

In the whole group, most patients were White (26,839; 78%) and older than 60 years (23,830; 69.2%), had the adenocarcinoma histological type (31,239; 90.7%), moderately differentiated tumors (25,438; 73.9%), LNH ≥ 12 (30,870; 89.7%), and LODDS stage 1 (21,981; 63.8%) and were CEA negative (22,289; 64.7%) and tumor deposit negative (32,106; 93.2%). Moreover, 67.1% of patients across the entire study population did not undergo chemotherapy. TNM stage I, II, and III tumors accounted for 21.9%, 39.0%, and 39.1% of all cases, respectively. The 1-, 3-, and 5-year CSS rates were 95.0%, 87.8%, and 82.7% for all patients, respectively, with a mean follow-up time of 72.7 months. The 1-, 3-, and 5-year OS rates were 91.8%, 81.2%, and 72.3% for all patients, respectively, with a mean follow-up time of 66.4 months.

### Independent prognostic factors in stage I–III colon cancer patients

According to the results based on the univariate Cox regression analysis, 13 variables, namely, sex, age at diagnosis, primary tumor site, histological type, pathological grade, adjuvant chemotherapy, LNH, LODDS stage, tumor size, CEA level, marital status, T stage, and N stage, were associated with CSS and OS (Tables 2, 3). In the multivariate Cox regression analysis, twelve parameters, namely, age at diagnosis, primary tumor site, histological type, pathological grade, adjuvant chemotherapy, LNH, LODDS stage, tumor size, CEA level, marital status, T stage, and N stage, were defined as independent prognostic factors predicting the CSS of stage I–III colon cancer patients (Table 2). All thirteen comparable variables (i.e., sex, age at diagnosis, primary tumor site, histological type, pathological grade, adjuvant chemotherapy, LNH, LODDS stage, tumor size, CEA level, marital status, T stage, and N stage) were defined as independent prognostic factors predicting the OS of stage I–III colon cancer patients (Table 3).

**Table 2:** Univariable and multivariable Cox regression model analyses of cause-specific survival in nomogram cohort

Variable	Univariable analysis			Multivariable analysis		
	HR	95% CI	P-value	HR	95% CI	P-value
Gender			0.002			0.066
Female	1			1		
Male	0.909	0.857–0.965		1.058	0.965–1.054	
Race			0.985			
White	1					
Other^a^	1.001	0.907–1.105				
Year of diagnosis			0.445			
2010–2012	1					
2013–2015	0.976	0.915–1.040				
Age at diagnosis			< 0.001			< 0.001
< 60	1			1		
≥ 60	1.913	1.781–2.056		1.718	1.591–1.856	
Tumor site			< 0.001			0.003
Right	1			1		
Left	0.825	0.778–0.875		0.912	0.857–0.970	
Histological subtype			< 0.001			0.001
AD	1			1		
MAD	1.251	1.135–1.378	< 0.001	0.983	0.891–1.085	0.738
SRCC	4.042	3.368–4.851	< 0.001	1.444	1.195–1.746	< 0.001
Pathological grade			< 0.001			< 0.001
Grade I	1			1		
Grade II	1.447	1.259–1.664	< 0.001	1.080	0.939–1.243	0.282
Grade III	2.867	2.477–3.319	< 0.001	1.350	1.162–1.568	< 0.001
Grade IV	3.704	3.091–4.439	< 0.001	1.650	1.372–1.984	< 0.001
T stage			< 0.001			< 0.001
T1	1			1		
T2	2.022	1.590–2.573	< 0.001	1.741	1.366–2.218	< 0.001
T3	5.497	4.429–6.822	< 0.001	3.579	2.864–4.472	< 0.001
T4a	14.594	11.686–18.226	< 0.001	7.283	5.775–9.186	< 0.001
T4b	17.479	13.929–21.933	< 0.001	9.477	7.467–12.029	< 0.001
N stage			< 0.001			< 0.001
N0	1			1		
N1	2.418	2.257–2.591	< 0.001	2.090	1.774–2.461	< 0.001
N2	4.679	4.437–5.125	< 0.001	3.220	2.678–3.872	< 0.001
Chemotherapy			< 0.001			< 0.001
No	1			1		
Yes	1.142	1.076–1.213		0.498	0.465–0.534	
LNH			< 0.001			< 0.001
< 12	1			1		
≥ 12	0.669	0.617–0.726		0.582	0.535–0.634	
LODDS stage			< 0.001			< 0.001
LODDS 1	1			1		
LODDS 2	2.689	2.528–2.861	< 0.001	1.290	1.097–1.517	0.002
LODDS 3	7.359	6.64–8.156	< 0.001	2.005	1.647–2.442	< 0.001
LODDS 4	21.948	16.814–28.649	< 0.001	4.274	3.109–5.876	< 0.001
Tumor size			< 0.001			0.038
< 4	1			1		
≥ 4	1.781	1.671–1.899		1.075	1.004–1.152	
CEA			< 0.001			< 0.001
Negative	1			1		
Positive	2.292	2.163–2.429		1.575	1.484–1.671	
Marriage status			< 0.001			< 0.001
Married	1			1		
Single	1.323	1.199–1.459	< 0.001	1.197	1.085–1.321	< 0.001
Separated/Divorced	1.397	1.285–1.519	< 0.001	1.298	1.193–1.493	< 0.001
Widowed	2.084	1.937–2.243	< 0.001	1.470	1.362–1.587	< 0.001

*HR* hazard ratio, *CI* confidence interval, *AD* adenocarcinoma, *MAD* mucinous adenocarcinoma, *SRCC* signet ring cell carcinoma, *LNH* lymph nodes harvested, *LODDS* log of odds between the number of positive lymph node and the number of negative lymph node, *CEA* carcinoembryonic antigen, *TNM* tumor-node-metastasis

^a^Includes Black, Native American, Asian, Pacific Islander and Unknown

**Table 3:** Univariable and multivariable Cox regression model analyses of overall survival in nomogram cohort

Variable	Univariable analysis			Multivariable analysis		
	HR	95% CI	P-value	HR	95% CI	P-value
Gender			0.017			0.001
Female	1			1		
Male	1.005	1.002–1.036		1.248	1.190–1.309	
Race			0.097			
White	1					
Other^a^	0.970	0.935–1.006				
Year of diagnosis			0.082			
2010–2012	1					
2013–2015	0.957	0.910–1.006				
Age at diagnosis			< 0.001			< 0.001
< 60	1			1		
≥ 60	2.724	2.560–2.899		2.228	2.086–2.379	
Tumor site			< 0.001			< 0.001
Right	1			1		
Left	0.753	0.719–0.789		0.865	0.825–0.908	
Histological subtype			< 0.001			0.001
AD	1			1		
MAD	1.260	1.170–1.358	< 0.001	1.024	0.949–1.104	0.540
SRCC	2.947	2.500–3.474	< 0.001	1.403	1.184–1.663	< 0.001
Pathologic grade			< 0.001			< 0.001
Grade I	1			1		
Grade II	1.199	1.090–1.320	< 0.001	1.029	0.934–1.134	0.557
Grade III	1.923	1.734–2.134	< 0.001	1.206	1.083–1.342	0.001
Grade IV	2.371	2.064–2.724	< 0.001	1.418	1.231–1.634	< 0.001
T stage			< 0.001			< 0.001
T1	1			1		
T2	1.462	1.292–1.654	< 0.001	1.317	1.162–1.493	< 0.001
T3	2.268	2.034–2.528	< 0.001	1.769	1.576–1.987	< 0.001
T4a	4.671	4.146–5.262	< 0.001	3.114	2.737–3.543	< 0.001
T4b	5.110	4.498–5.806	< 0.001	3.634	3.163–4.175	< 0.001
N stage			< 0.001			< 0.001
N0	1			1		
N1	1.533	1.456–1.615	< 0.001	1.646	1.444–1.876	< 0.001
N2	2.557	2.413–2.709	< 0.001	2.364	2.035–2.746	< 0.001
Chemotherapy			< 0.001			< 0.001
No	1			1		
Yes	0.733	0.697–0.771		0.429	0.404–0.455	
LNH			< 0.001			< 0.001
< 12	1			1		
≥ 12	0.674	0.633–0.717		0.621	0.581–0.663	
LODDS stage			< 0.001			< 0.001
LODDS 1	1			1		
LODDS 2	1.674	1.598–1.754	< 0.001	1.247	1.093–1.423	0.001
LODDS 3	3.941	3.602–4.312	< 0.001	1.853	1.570–2.188	< 0.001
LODDS 4	10.562	8.166–13.662	< 0.001	3.592	2.674–4.825	< 0.001
Tumor size			< 0.001			0.048
< 4	1			1		
≥ 4	1.446	1.379–1.517		1.050	1.004–1.102	
CEA			< 0.001			< 0.001
Negative	1			1		
Positive	1.943	1.858–2.032		1.515	1.446–1.587	
Marriage status			< 0.001			< 0.001
Married	1			1		
Single	1.318	1.220–1.423	< 0.001	1.266	1.171–1.367	< 0.001
Separated/divorced	1.366	1.279–1.460	< 0.001	1.394	1.304–1.491	< 0.001
Widowed	2.375	2.248–2.510	< 0.001	1.756	1.654–1.865	< 0.001

*HR* hazard ratio, *CI* confidence interval, *AD* adenocarcinoma, *MAD* mucinous adenocarcinoma, *SRCC* signet ring cell carcinoma, *LNH* lymph nodes harvested, *LODDS* log of odds between the number of positive lymph node and the number of negative lymph node, *CEA* carcinoembryonic antigen, *TNM* tumor-node-metastasis

^a^Includes Native American, Asian, Pacific Islander and Unknown

### Construction and validation of the prognostic prediction nomogram

**Fig. 2:**
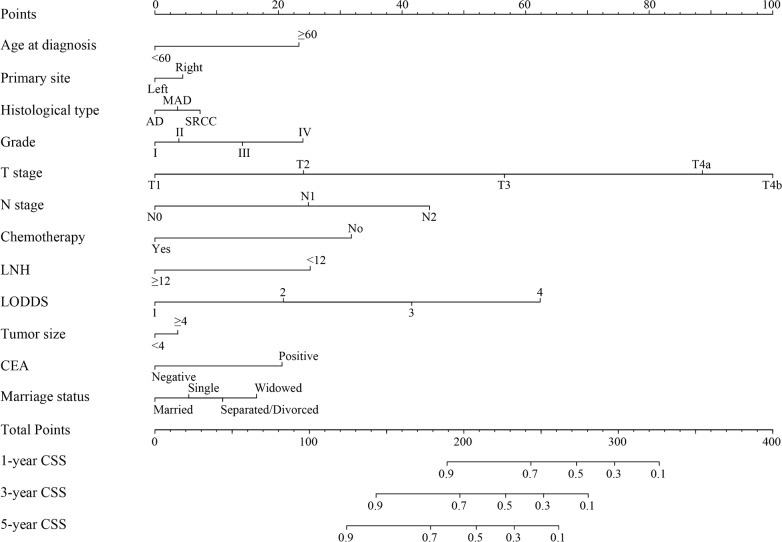
Nomogram convey the results of prognostic models using twelve clinicopathological characteristics to predict cause-specific survival of patients with stage I–III colon cancer

**Fig. 3:**
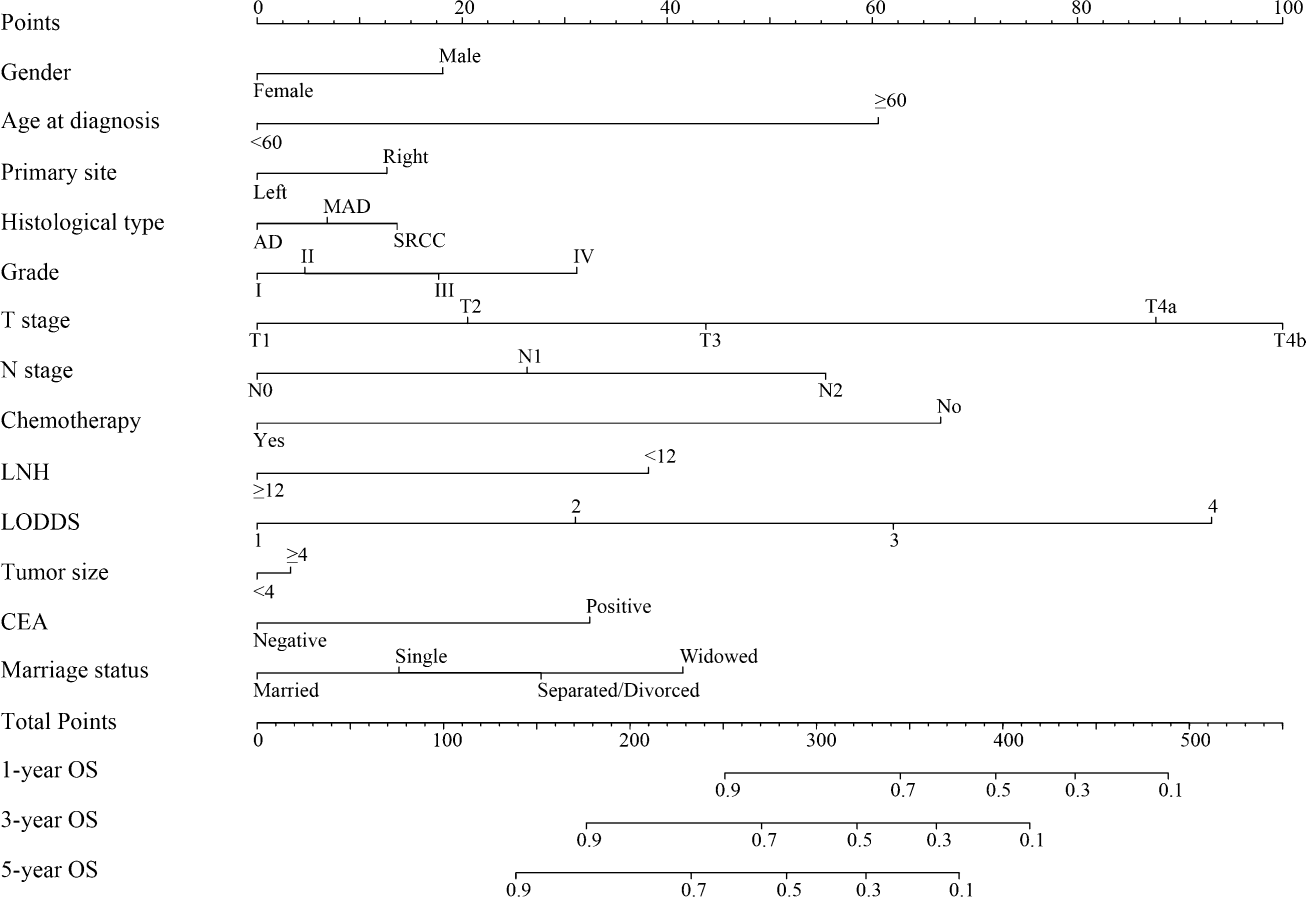
Nomogram convey the results of prognostic models using thirteen clinicopathological characteristics to predict overall survival of patients with stage I–III colon cancer

Considering the results of the multivariable Cox regression analysis for CSS and OS, all of the significant variables were used to create the nomogram for CSS and OS. The prognostic nomogram for 1-, 3-, and 5-year CSS is shown in Fig. 2. The prognostic nomogram for 1-, 3-, and 5-year OS is shown in Fig. 3. By summing the scores associated with each variable and projecting total scores to the bottom scale, the probabilities can be estimated for 1-, 3-, and 5-year CSS and OS.

**Fig. 4:**
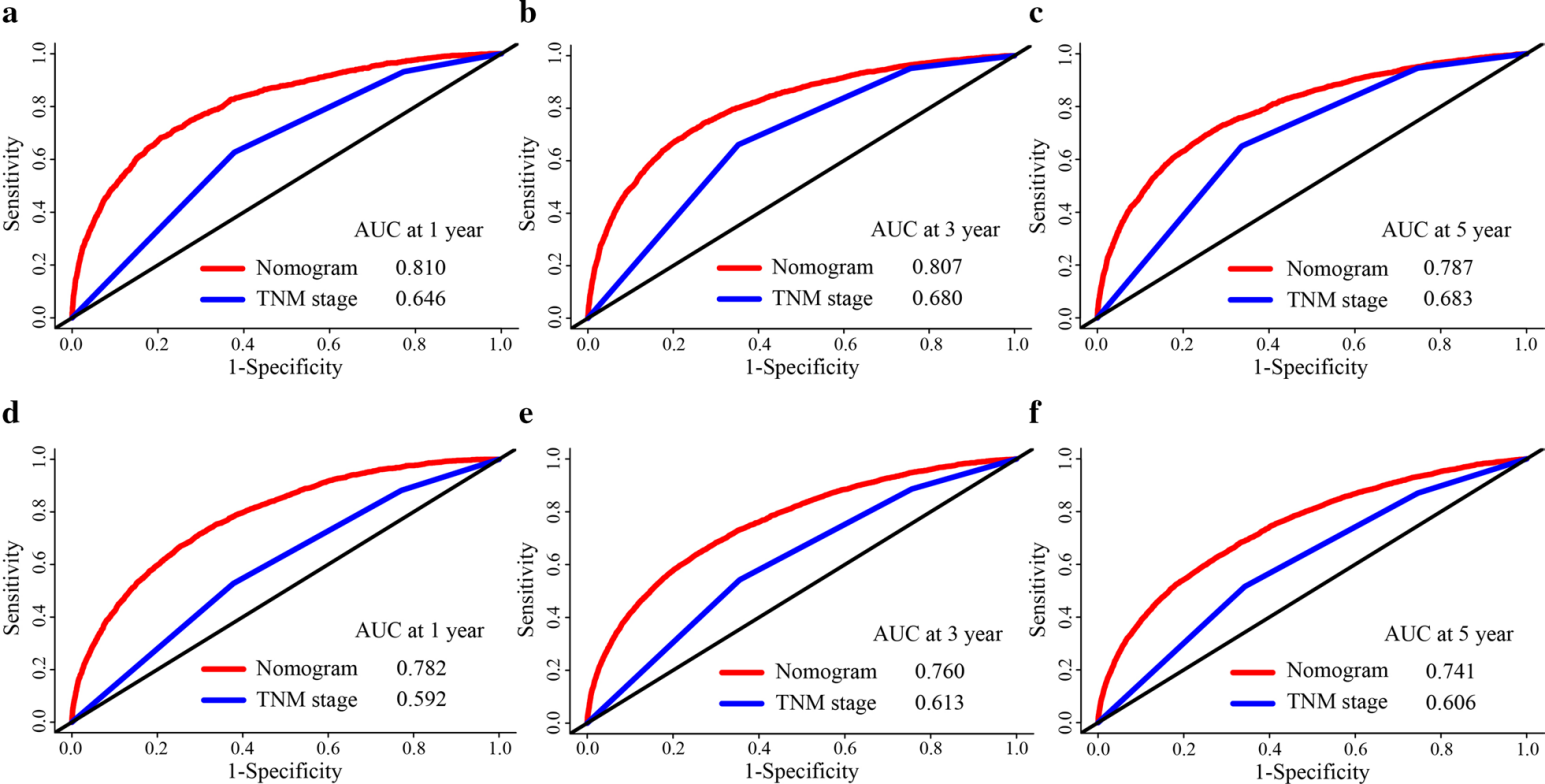
**a** AUC values of ROC predicted 1-year cause-specific survival rates of Nomogram and TNM stage. **b** AUC values of ROC predicted 3-year cause-specific survival rates of Nomogram and TNM stage. **c** AUC values of ROC predicted 5-year cause-specific survival rates of Nomogram and TNM stage. **d** AUC values of ROC predicted 1-year overall survival rates of Nomogram and TNM stage. **e** AUC values of ROC predicted 3-year overall survival rates of Nomogram and TNM stage. **f** AUC values of ROC predicted 5-year overall survival rates of Nomogram and TNM stage

**Fig. 5:**
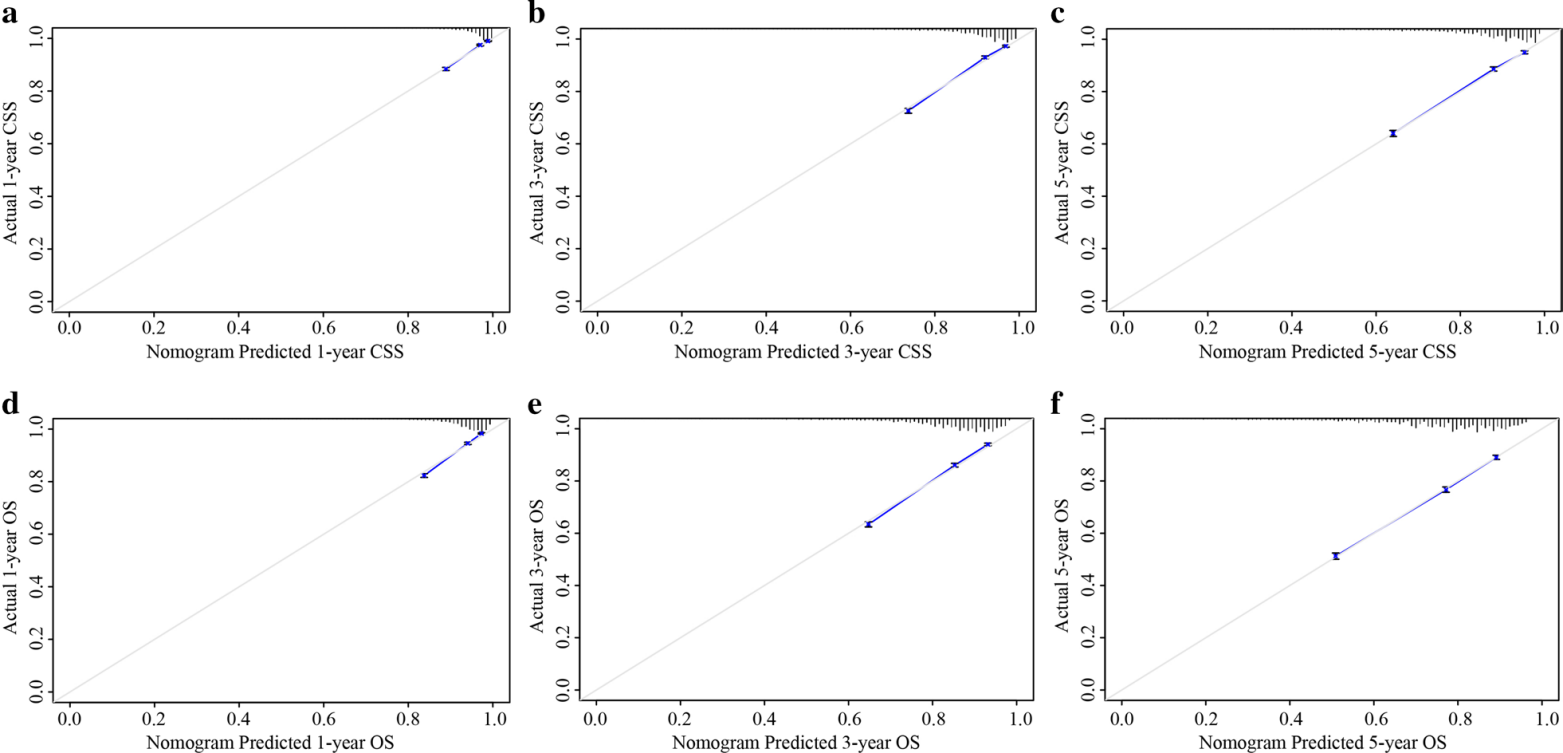
**a** The calibration curve for predicting patients’ cause-specific survival at 1-year. **b** The calibration curve for predicting patients’ cause-specific survival at 3-year. **c** The calibration curve for predicting patients’ cause-specific survival at 5-year. **d** The calibration curve for predicting patients’ overall survival at 1-year. **e** The calibration curve for predicting patients’ overall survival at 3-year. **f** The calibration curve for predicting patients’ overall survival at 5-year

C-index values and ROC curves are ordinarily used to evaluate the discriminatory power of a nomogram. The C-indexes for the prediction of CSS and OS were 0.78 (95% CI 0.77–0.80) and 0.74 (95% confidence interval (CI) 0.73–0.75), respectively. To confirm that the nomogram had higher efficacy in predicting the prognosis of stage I–III colon cancer patients than TNM stage, time-dependent ROC analyses at 1, 3, and 5 years were conducted. The 1-, 3-, and 5-year AUC values of the nomogram for the prediction of CSS were 0.81, 0.807, and 0.787, respectively, compared with 0.646, 0.680, and 0.683, respectively, for the AUC values of TNM stage (Fig. 4a–c). In addition, the 1-, 3-, and 5-year AUC values of the nomogram for the prediction of OS were 0.782, 0.76, and 0.741, respectively, compared with 0.592, 0.613, and 0.606, respectively, for the AUC values of TNM stage (Fig. 4d–f). In addition, calibration curves for the nomogram showed no deviations from the reference line, which indicating a high degree of credibility (Fig. 5a–f).

**Fig. 6:**
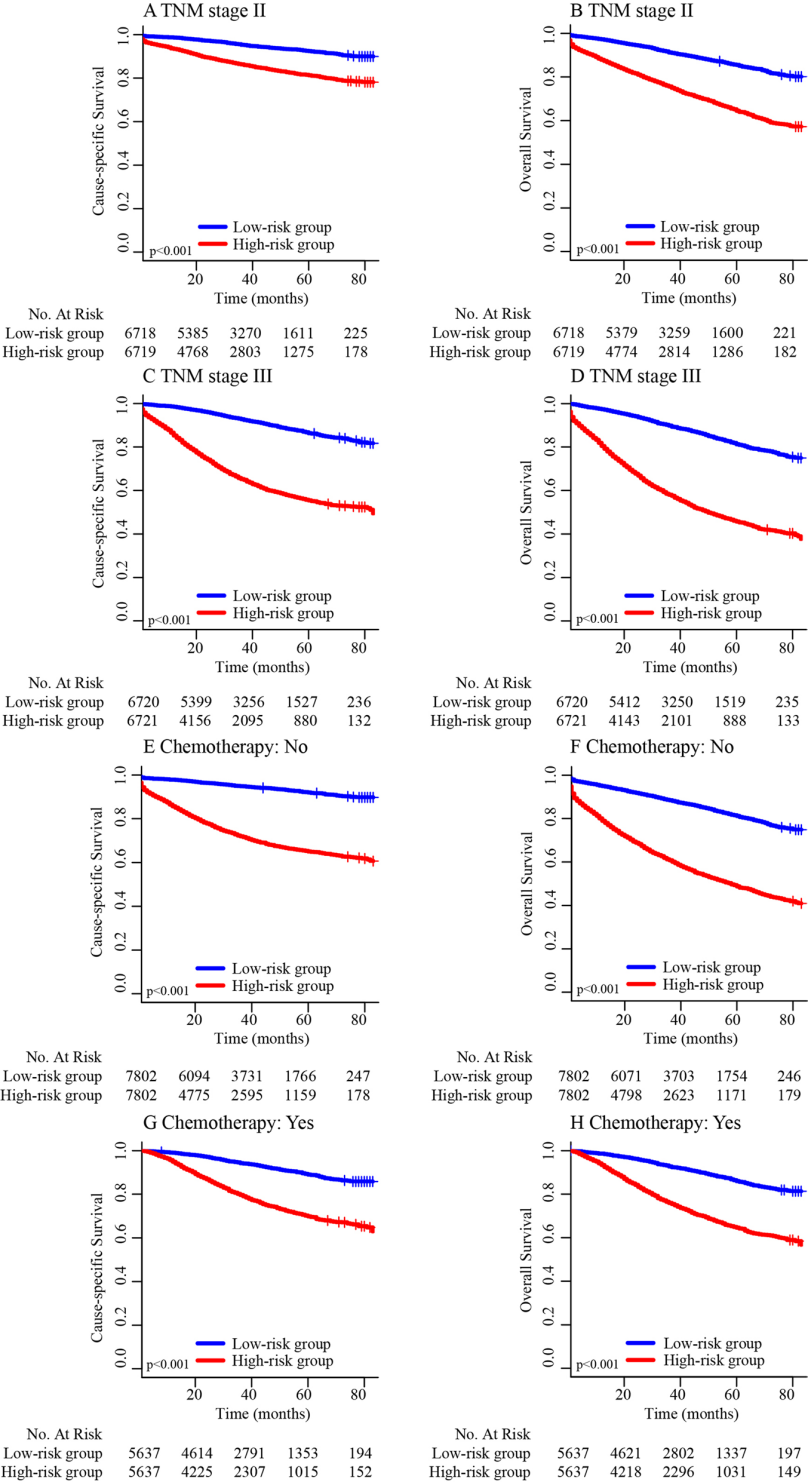
**a** Kaplan–Meier estimated cause-specific survival in patients with TNM stage II colon cancer stratified by the nomogram risk score. **b** Kaplan–Meier estimated overall survival in patients with TNM stage II colon cancer stratified by the nomogram risk score. **c** Kaplan–Meier estimated cause-specific survival in patients with TNM stage III colon cancer stratified by the nomogram risk score. **d** Kaplan–Meier estimated overall survival in patients with TNM stage III colon cancer stratified by the nomogram risk score. **e** Kaplan–Meier estimated cause-specific survival in stage II–III colon cancer patients without chemotherapy stratified by the nomogram risk score. **f** Kaplan–Meier estimated overall survival in stage II–III colon cancer patients without chemotherapy stratified by the nomogram risk score. **g** Kaplan–Meier estimated cause-specific survival in stage II–III colon cancer patients with chemotherapy stratified by the nomogram risk score. **h** Kaplan–Meier estimated overall survival in stage II–III colon cancer patients with chemotherapy stratified by the nomogram risk score

The clinically and statistically significant prognostic performance of the nomogram based on the entire group of patients and risk scores was validated by a stratified analysis, which suggested that the nomogram could be used to clinically and statistically predict the prognosis of patients with stage II (Fig. 6a, b), and stage II–III colon cancer with or without adjuvant chemotherapy (Fig. 6e–h).

### Clinical value of the nomogram

**Fig. 7:**
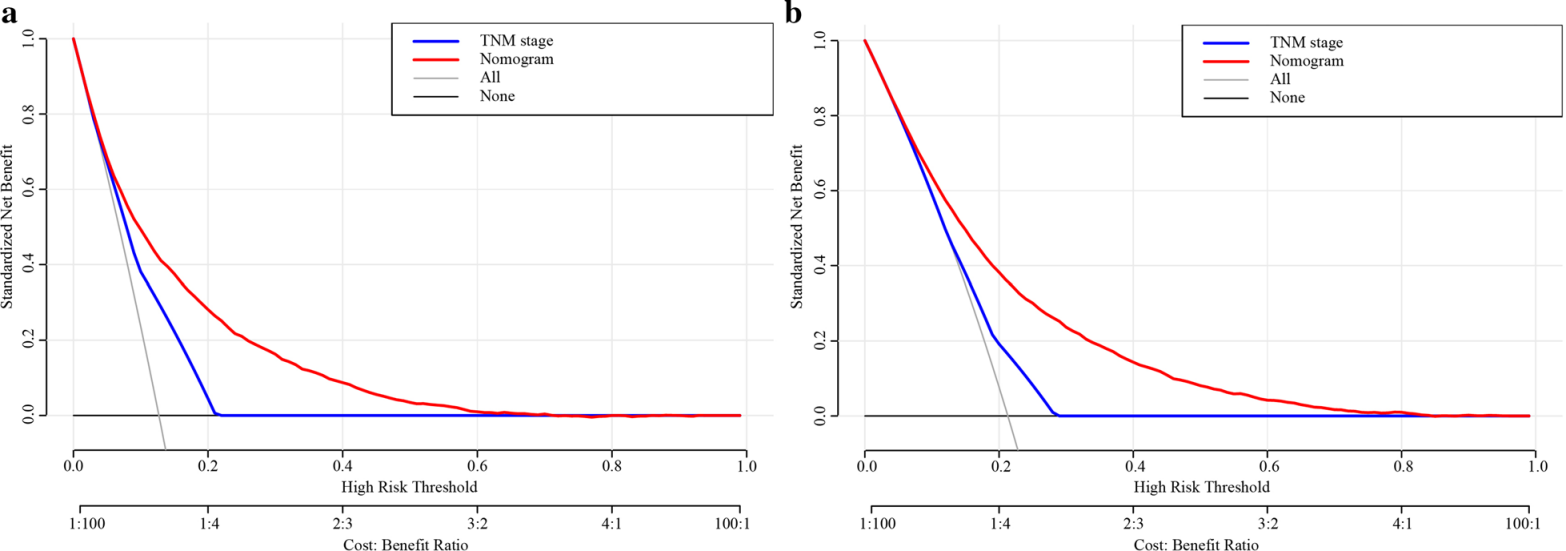
**a** Decision curve analysis of the nomogram and TNM stage for the cause-specific survival prediction of stage I–III colon cancer patients. **b** Decision curve analysis of the nomogram and TNM stage for the overall survival prediction of stage I–III colon cancer patients

DCA is a novel method used to evaluate alternative prognostic strategies and has advantages over the AUC. DCA curves for the novel nomogram and TNM stage are presented in Fig. 7. Compared with the TNM staging system, the DCA of the nomogram has higher net benefits, indicating that it has better clinical application value than TNM stage.

### Prognostic nomogram for risk stratification

**Fig. 8:**
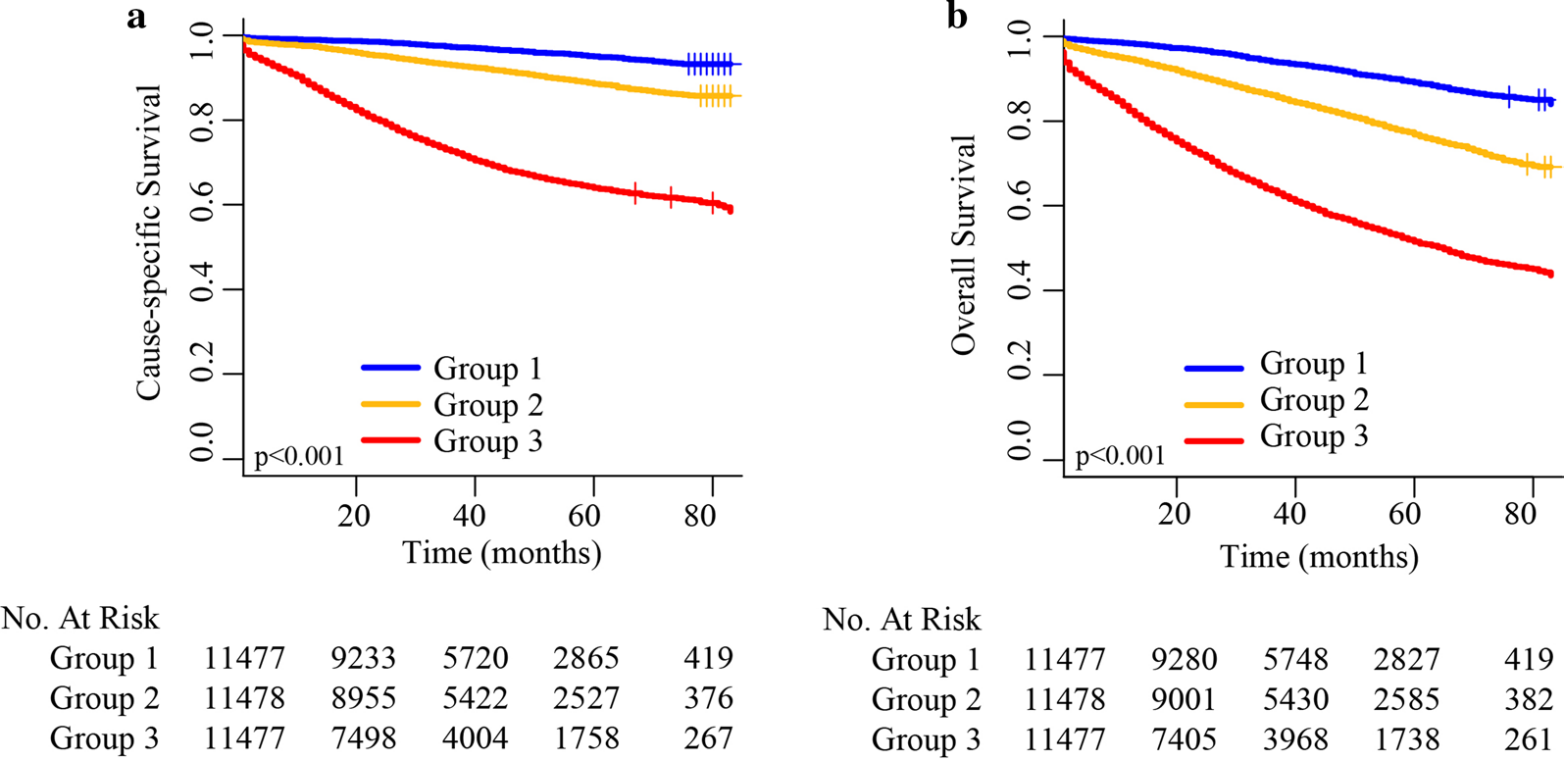
**a** Cause-specific survival in the subgroups according to a tertiles of the total score. **b** Overall survival in the subgroups according to a tertiles of the total score

By regrouping all patients in the CSS and OS cohorts into three subgroups based on the total scores, the cut-off values were defined, and each group represents a distinct prognosis. The Kaplan–Meier survival curves were subsequently delineated and are shown in Fig. 8. In the CSS cohort, Group 1 (low-risk group) had the highest 5-year CSS rate of 95.0%, followed by Group 2 (moderate-risk group; 88.6%) and Group 3 (high-risk group 64.0%). In the OS cohort, Group 1 (low-risk group) had the highest 5-year OS rate of 89.1%, followed by Group 2 (moderate-risk group 76.8%) and Group 3 (high-risk group 51.5%). A significant statistical distinction in survival outcomes was observed between the three groups.

## Discussion

Through this study, a nomogram merging clinicopathological parameters with the TNM staging system was built to assess the definite 1-, 3-, and 5-year CSS and OS probabilities of stage I–III colon cancer patients. The behavior of the nomogram (i.e., discrimination and calibration) was verified. From the perspective of clinical influence, the nomogram had a wide range of threshold probabilities. From the perspective of ROC curve analysis and DCA, the nomogram showed better predictive accuracy and prognostic value in stage I–III colon cancer compared to the current TNM staging system. Moreover, the nomogram was competent to divide patients with stage I–III colon cancer into low-, moderate-, and high-risk groups, which indicates that the nomogram can be utilized as a conventional equipment in predicting the prognosis of stage I–III colon cancer.

In the present study, it was found that the number of young individuals diagnosed with colon cancer has increased. Previous research has revealed that age is an independent prognostic factor of stage I–III colon cancer patients, with a younger age indicating more pronounced outcomes [6]. In addition, a considerable prognostic factor certified by this study was CEA, which is a well-established biomarker for colon cancer recommended by both the American Society of Clinical Oncology (ASCO) and the European Group on Tumor Markers (EGTM) [18–20]. Preoperative CEA levels were used to predict prognosis, and routine CEA monitoring during the postoperative follow-up was used to monitor local relapse and distant metastases after colon cancer surgery. As this nomogram showed, stage I–III colon cancer patients with high CEA levels tended to have significantly poor CSS and OS rates. In addition, left-sided colon cancers (LCCs) and right-sided colon cancers (RCCs) are thought to have different embryological origins [21]. Various differences, such as anatomical structure, function, morphological characteristics, and histochemical reactions, exist between the two. Patients with LCC have a significantly better prognosis than those with RCC in terms of OS, which was indicated by this research. In addition, tumor size [5] was validated as an independent factor for OS in patients with colorectal adenocarcinoma of infiltrative and ulcerative types in a previous study. This research suggested that large tumors led to a poor prognosis.

Whether adjuvant chemotherapy is suitable for stage I–III colon cancer remains controversial. According to the NCCN guidelines, it is recommended that patients with stage II colon cancers with risk factors and stage III colon cancers accept adjuvant chemotherapy [22, 23]. In this study, histological differentiation, grade, right colon, LNH less than 12, LODDS, tumor size, marital status, T stage, and N stage were identified as independent risk factors for stage I–III colon cancer [14]. Histological differentiation was identified as an important feature to evaluate the benefit of adjuvant chemotherapy in a previous study [24]. This nomogram proved that low histological differentiation was associated with a poor prognosis. Low histological grade was considered among the adverse histopathological factors associated with an unfavorable clinical course of colon cancer. A previous study demonstrated that tumor location was associated with prognosis in colon cancer patients [21]. Furthermore, the appropriate staging of colon cancer requires at least 12 lymph nodes to be sampled, as recommended by the NCCN guidelines. Relevant research indicated that stage I–III colon cancer patients with LNH less than 12 tended to have shorter CSS and OS than those with LNH more than 12, which corroborated the results of this nomogram [25].

Some scholars have trusted that the LODDS is a more accurate method for predicting the prognosis of patients with colon cancer after an operation than the N stage. The LODDS was defined as follows: log((number of positive lymph nodes + 0.05)/(number of negative nodes + 0.05)). The LODDS range in this research was − 3.256 to 2.858. The LODDS system was divided into four levels to determine the LODDS status: LODDS1 (LODDS ≤ − 1.5), LODDS2 (− 1.5 ≤ LODDS < 0), LODDS3 (0 ≤ LODDS < 1.5), and LODDS4 (LODDS ≥ 1.5) [26, 27]. This nomogram showed that a high LODDS status was related to poor survival outcomes.

Marital status is another independent prognostic factor for survival in colon cancer. Previous research showed that being married was associated with better outcomes of colon cancer patients, but unmarried colon cancer patients, including single, separated, divorced, and widowed patients, were at a greater risk of mortality [15], which was reproduced in this research. Our nomogram shows that separated, divorced, and widowed patients were associated with a greater risk of mortality.

However, this study still had some limitations. First, treatment information except for surgery was not available in the SEER database and was thus not incorporated into our analysis. Second, the SEER database is devoid of variables such as detailed histological information, mode of presentation, and ECOG prognostic scores and lacks 90% of biomarker expression states (e.g., RAS, BRAF, PIK3CA and genes involved in DNA mismatch repair, which have been proven to predict survival). Last, this study did not contain any external validation cohort. Additional prospective data and the incorporation of other factors are encouraged to improve this model.

## Conclusion

In conclusion, we established and validated a nomogram for predicting CSS and OS probabilities in stage I–III colon cancer patients. The simple nomogram had sufficient discriminatory and calibration capability in addition to exceptional clinical effectiveness and could be an easy-to-use tool for clinicians to promote a personalized postoperative prognostic assessment and to identify treatment strategies for patients with stage I–III colon cancer.

## Data Availability

The dataset used during the study are available from the corresponding author on a reasonable request.
